# The “Neuro-Glial-Vascular” Unit: The Role of Glia in Neurovascular Unit Formation and Dysfunction

**DOI:** 10.3389/fcell.2021.732820

**Published:** 2021-09-27

**Authors:** Elisabeth C. Kugler, John Greenwood, Ryan B. MacDonald

**Affiliations:** Institute of Ophthalmology, Faculty of Brain Sciences, University College London, London, United Kingdom

**Keywords:** astrocytes, brain, Müller glia, neurovascular unit, retina

## Abstract

The neurovascular unit (NVU) is a complex multi-cellular structure consisting of endothelial cells (ECs), neurons, glia, smooth muscle cells (SMCs), and pericytes. Each component is closely linked to each other, establishing a structural and functional unit, regulating central nervous system (CNS) blood flow and energy metabolism as well as forming the blood-brain barrier (BBB) and inner blood-retina barrier (BRB). As the name suggests, the “neuro” and “vascular” components of the NVU are well recognized and neurovascular coupling is the key function of the NVU. However, the NVU consists of multiple cell types and its functionality goes beyond the resulting neurovascular coupling, with cross-component links of signaling, metabolism, and homeostasis. Within the NVU, glia cells have gained increased attention and it is increasingly clear that they fulfill various multi-level functions in the NVU. Glial dysfunctions were shown to precede neuronal and vascular pathologies suggesting central roles for glia in NVU functionality and pathogenesis of disease. In this review, we take a “glio-centric” view on NVU development and function in the retina and brain, how these change in disease, and how advancing experimental techniques will help us address unanswered questions.

## Introduction

**FIGURE 1:**
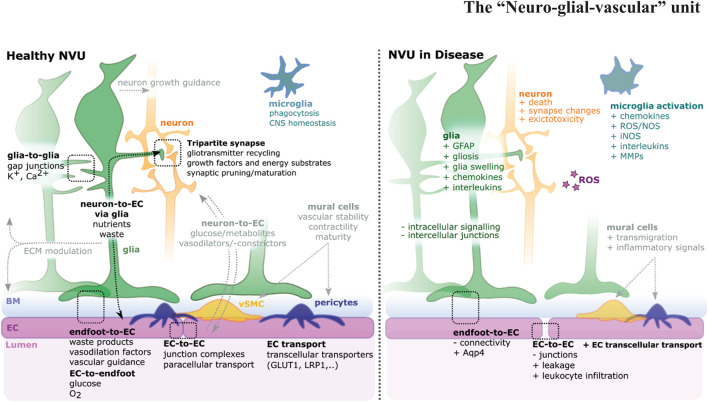
Schematic of the neurovascular unit (NVU) in health and disease. The NVU is a hetero-cellular complex formed by glia, neurons, vSMCs, pericytes, microglia, and blood vessels, that form the blood-brain barrier (BBB) and blood retina barrier (BRB). Glia cells (green) impact neurons (orange), endothelial cells (ECs) (magenta), and each other. For NVU functionality, various direct (i.e., glia-to-glia, tripartite synapse, endfoot-to-EC, EC-to-endfoot) and indirect (i.e., neuron-to-EC *via* glia, neuron-to-EC *via* mural cells, microglia) pathways need to be considered. Upon disease multi-level changes are observed, including altered cell shapes, function, and interactions [see also (Lécuyer et al., 2016; Jha et al., 2018)]. NVU component changes include gliosis, neuron death, EC-connectivity changes, mural cell transmigration, and microglia activation.

The brain and retina, which constitute the central nervous system (CNS), are highly complex tissues, requiring high levels of energy for function and tight control for health. To achieve this, they contain a specialized vasculature that controls parenchymal homeostasis, transport of metabolites, and confers, in part, so-called immune privilege (Forrester et al., 2018; Taylor and Ng, 2018). Most importantly, the bi-directional movement of molecules across the blood-tissue barriers is strictly controlled to maintain CNS health and brain function. For many decades the focus of this regulatory capacity lay at the endothelial cell (EC), the predominant cell of the blood-brain barrier (BBB), but more recently the wider neurovascular unit (NVU) has been recognized as providing functionality. The NVU is a complex multi-hetero-cellular structure of EC, neurons, glia, smooth muscle cells (SMCs), pericytes, and extracellular matrix (ECM). Together, these components regulate blood flow and metabolism, thus allowing the controlled exchange of nutrients and metabolic waste products (Hawkins and Davis, 2005; Lok et al., 2007). To meet the high metabolic demand of the CNS, particularly in response to an intensification of physical or mental activity, increased neuronal activity leads to subsequent changes in cerebral blood flow (functional hyperemia), in a process called neurovascular coupling. While neurons can directly regulate this system (McConnell et al., 2017), glial cells are often in direct physical contact with the vasculature and neurons, thus are critically positioned to interface between these cellular components where they may contribute to the relay of information and act as a modulator of such crosstalk. Additionally, NVU components are crucial for brain protection and homeostasis as ECs form the BBB, and glia physically ensheathing the blood vessels are seen as a secondary barrier (Kutuzov et al., 2018). With this close physical interaction between ECs and glia, nutrients required for CNS function are delivered from the blood vessels to neurons mainly *via* glia cells (Hurley et al., 2015), while waste compounds are passed *via* glial cell to microglia or back into the bloodstream (Marina et al., 2018). Dysfunction of the NVU is characterized by dysregulation of neurovascular coupling, neuron death, gliosis, microglia activation, mural cell transmigration, and BBB breakdown (Zlokovic, 2005; Willis, 2011). Dysfunction of the BBB is associated with increased vascular leakage, transcellular transport, immune cell infiltration, and reduction of intercellular junctions (Figure 1). Accordingly, glial cells and other NVU cells work closely together to maintain CNS function and maintenance.

In this review, we discuss glia cell types and their role in the NVU, by examining glia specializations to support neurons, the vasculature, and neuro-vascular interactions in the NVU. Lastly, we will highlight how rapidly improving techniques and tools will help us answer pressing outstanding questions in the field. While this review is not meant to be an exhaustive list of the many types of glia within the CNS, we aim to highlight their importance for the development, function, and dysfunction of the NVU in the brain and retina.

## Components of the Neurovascular Unit

**FIGURE 2:**
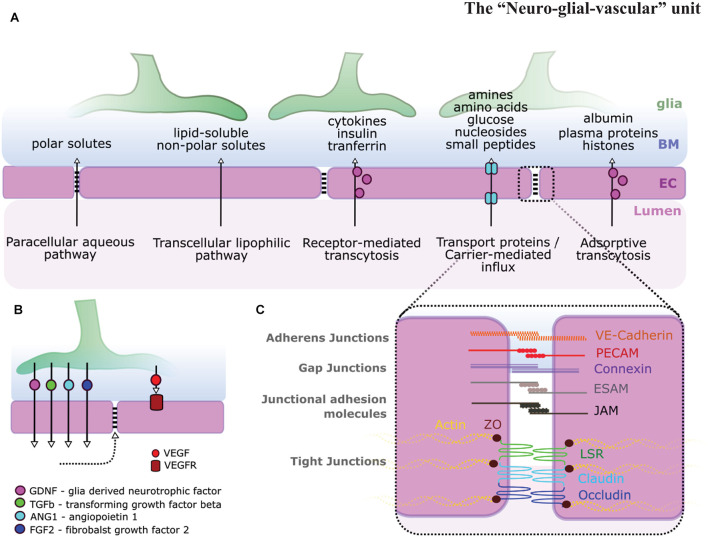
Glia-endothelial interactions. **(A)** Pathways across the BBB and BRB allow for the transport of various types of molecules. **(B)** Glial signaling impacts ECs [i.e., glial-derived neurotrophic factor (GDNF), transforming growth factor β (TGF-β), Ang1, fibroblast growth factor 2 (FGF2), and vascular endothelial growth factor (VEGF)] and in turn BBB stability (dotted arrow). **(C)** BBB stability is highly dependent on EC inter-cellular junction integrity including adherens junctions, gap junctions, junctional adhesion molecules, and tight junctions adapted from Abbott et al. (2006); Malik and Di Benedetto (2018).

The complex interaction between NVU cells requires each cellular component to operate in a complex and coordinated manner to ensure homeostatic control of the BBB and blood retina barrier (BRB). Each component exhibits specialized features that are critical to the overall maintenance of NVU function (Figure 1). Briefly, ECs form a single-layer lining of tubular blood vessels, which are specialized depending on the vascular bed in which they are situated (Chico and Kugler, 2021). At the BBB/BRB, ECs exhibit reduced pinocytosis/transcytosis (O’Brown et al., 2019; Villaseñor et al., 2019), increased expression of tight junction molecules, such as claudins, occludin, or zonula occludens 1 (ZO-1; Figure 2A; Fanning et al., 1999; Nitta et al., 2003), and exclude free transport of substances over 400kDa (Pardridge, 2001). Mural cells, which constitute pericytes and vascular smooth muscle cells (vSMCs), are positioned in the basement membrane shared with ECs, maintain vascular stability, provide structural support for blood vessels, govern vasodilation/constriction (Tong et al., 2021), as well as contribute to NVU function by BBB maintenance (Armulik et al., 2010; Bell et al., 2010; Henshall et al., 2015). Neurons in the NVU transduce signals and control local cerebral blood flow directly, such as *via* nitric oxide (NO), as well as indirectly *via* glia cells, such as *via* arachnoid acid or potassium (K^+^) (Attwell et al., 2010). Additionally, neuronal activity itself shapes vascular and BBB formation *via* levels of neurotransmitter release (Lacoste et al., 2014; Whiteus et al., 2014). Glial cells physically ensheath blood vessels with their endfeet, creating the semi-permeable glia limitans (Kutuzov et al., 2018). Importantly, glia physically connect vessels to neurons (Zonta et al., 2003), modulate neurotransmission, and impact neurogenesis (Argente-Arizón et al., 2017; Falk and Götz, 2017). Microglia, macrophages, and perivascular macrophages (PVM) play roles in phagocytosis and the CNS inflammatory response, ensuring CNS maintenance and health (Guillemin and Brew, 2004; Serrats et al., 2010). The vascular basement membrane, which encompasses blood vessels, acts as a passage for fluid transport (Morris et al., 2016), while the perivascular basal laminae and ECM molecules support the glio-vascular interface (Hoddevik et al., 2020). Together, these components form a spatially and functionally integrated NVU with bidirectional communication, namely neuro-vascular-coupling and vascular-neuro-coupling. However, the precise mechanisms of the diverse roles of glial cells in NVU form, maintenance and function remain unclear. Answering these fundamental questions will be of particular importance as the NVUs’ main role is considered neurovascular coupling, but indeed various other aspects such as integration of signaling, metabolism, and homeostasis occur across NVU components and thus must also be considered.

## Overview of Glial Cells Types and the Neurovascular Unit

Glia were originally described as scaffolds providing structural support and maintaining biophysical integrity (Virchow, 1856), making their role in supporting the structure and biophysical integrity of the CNS their most widely described function (Losada-Perez, 2018). However, glia are now being increasingly appreciated for their many other functional and regulatory roles, such as neurotransmission, BBB function, and controlling immunity (Table 1). To fulfill the variety of specialized functions required, glia cells are highly specialized according to each CNS region with respect to proteomic signatures, electrophysiology, Ca^2+^ signaling, morphology, and proximities to synapses (Tsai et al., 2012; Molofsky et al., 2014; Ben Haim and Rowitch, 2017; Chai et al., 2017). Moreover, even within morphological groups, such as astrocytes, there is heterogeneity of cell types and their pathological responses (John Lin et al., 2017; Hasel et al., 2021) that may also reflect regional differences in the structure and function of the NVU.

**TABLE 1:** Summary of glia cell types, function, shape, and markers.

**Glia cell type**	**Functions**	**Shape**
Radial glia	o Generate glia and neurons in development (Rakic, 2003)	Bipolar
	o Stem cells and BBB maintenance in adulthood (Sharif et al., 2018)	
Astrocytes	o Control neurotransmitters as well as ionic and osmotic homeostasis (Simard and Nedergaard, 2004; Keaney and Campbell, 2015; Price et al., 2018)	Star-shaped
	o Regulate blood vessel diameters (Kimelberg, 2010)	
	o Act as angiogenic templates (O’Sullivan et al., 2017)	
Müller glia	o Retina-specific, species-specific glia cells (Cajal, 1995)	Apico-basal organization with 5 sub-domains (Wang et al., 2017)
Microglia	o CNS primary immune cells (Nimmerjahn et al., 2005)	Highly plastic, depending on activation state
Ependymal cells	o Line the brain ventricles, producing cerebrospinal fluid, and act as progenitors (Bigio, 2010; Furube et al., 2020)	Simple columnar shape
Oligodendrocytes	o Axon insulation and create myelin (Elbaz and Popko, 2019)	Ensheath axons

The most abundant and widespread glial cell type in the brain are astrocytes. Astrocytes are fivefold more numerous than neurons (Sofroniew and Vinters, 2010), with individual astrocytes contacting up to two million neuron synapses with elaborate morphologies (Oberheim et al., 2009). This high spatial correspondence between astrocytes and neurons is accompanied by astrocytes regulating neuron health by controlling neurotransmitters, such as glutamate or adenosine, as well as maintaining hydromineral brain homeostasis, such as Ca^2+^, Cl^–^, or water (Simard and Nedergaard, 2004; Keaney and Campbell, 2015; Price et al., 2018). In addition to contacting neurons, astrocytes also contact blood vessels, affecting local blood flow by regulating blood vessel diameters by vasoconstriction (e.g., by arachnoid acid) and vasodilation (e.g., by prostaglandins) (Kimelberg, 2010). Astrocytes also physically and functionally contribute to the BBB and its permeability for factors such as molecular traffic of glucose or proteins (Abbott et al., 2006). Thus, astrocytes are central to the function of a healthy functioning NVU, and overall CNS function.

In the retina, the principal glial cell type are Müller glia (MG), which contact blood vessels and neurons, fulfilling similar functions as astrocytes in the brain (Newman and Reichenbach, 1996; Subirada et al., 2018). The structure, morphology, and species-specific differences of retinal MGs are well described (Cajal, 1995) and MG exhibit at least five apico-basal domains that stretch from the apical stem around photoreceptors to the basal endfoot in the inner limiting membrane (Wang et al., 2017). However, MG are not just highly organized apico-basally, but also laterally to interact with other cells; this intercalation between cells is in a so-called tiled fashion, thereby contacting almost all cells within the retina (MacDonald et al., 2017; Wang et al., 2017). The retina is protected by two separate components of the BRB. The outer (oBRB) consists of retinal pigment epithelium and the inner (iBRB) is located at the level of the retinal capillaries. The latter is established by MGs and pericytes, with dysfunction being implicated in several retinal diseases such as diabetic retinopathy (DR; Cunha-Vaz et al., 2011; Frey and Antonetti, 2011; Díaz-Coránguez et al., 2017; Park et al., 2017). In the mammalian retina, there are also astrocytes that contact blood vessels and are pivotal as a structural growth template during angiogenesis, mainly *via* vascular endothelial growth factor (VEGF) and Hypoxia-inducible factor (HIF) pathways, following ganglion cell templates (O’Sullivan et al., 2017; Paisley and Kay, 2021). This suggests that MG and astrocytes may work together in the mammalian retina to influence the development of the NVU as well as to regulate its function. In humans, the retinal astrocytes are limited to the inner vascular plexus, while MG contact both plexi and are likely to induce the BRB functionality/maturity in the deep plexus, raising the likelihood for differential coupling between astrocytes and MG in the healthy and potentially diseased retina (Tout et al., 1993; Fruttiger, 2007; Ashraf et al., 2020). However, MG are the only glia found in the fovea, which is free of astrocytes, microglia, and vascular EC, suggesting that in some regions of the retina MG are sufficient to solely meet the functional needs required for high acuity vision (Reichenbach and Bringmann, 2020).

Besides the principal glial cells in the brain and retina (i.e., astrocytes and MG), other glial cells exist to fulfill crucial functions in the CNS. During development, progenitors, so-called radial glia, will divide to generate neurons and glia (Rakic, 2003), while also making contact with the vasculature where they contribute to the regulation of CNS angiogenesis. In certain CNS regions, radial glia persist into adulthood as stem cells, contributing to BBB maintenance *via* retinoic acid signaling (Sharif et al., 2018). Microglia are the primary immune cells in the CNS, surveying their environment and responding to insult, fulfilling roles in phagocytosis and inflammation where they express both pro-inflammatory (e.g., IL-1β) and anti-inflammatory (e.g., IL-10) molecules, with subsequent upregulation of factors such as Glial Fibrillary Acidic Protein (GFAP). Additionally, microglia-to-astrocyte crosstalk in response to glutamate plays a role in neuro-immune-interactions (Nimmerjahn et al., 2005; Macht, 2016), and microglia are required for normal neurogenesis, mostly by nerve growth factor (NGF) and tumor necrosis factor (TNF; Matejuk and Ransohoff, 2020). Another type of glia, called ependymal cells, line the brain ventricles, producing cerebrospinal fluid, and subpopulations acting as progenitors for astrocytes and oligodendrocytes (Bigio, 2010; Furube et al., 2020). Thus, there are several types of glia found within each CNS tissue, yet several pressing questions remain to understand the importance of glia in the NVU. These include what role do glial cells play in driving NVU formation during development, how are glial cells directed to contact and support multi-cellular units within the NVU, and what are the consequences of dysfunction of these glial components on NVU function.

## Glial Support for Neurons in the Neurovascular Unit

The fact that glia are a structurally integral part of the NVU physically linking the vasculature and neurons, emphasizes their functionality in BBB/BRB formation and CNS development.

Glia cells provide structural support to neuronal tissues for anisotropic mechanical tension (Nagashima et al., 2017), and loss of MG in the retina results in tearing of the tissue due to the loss of their biophysical support (MacDonald et al., 2015). Another biophysical role of glia is that they can swell, which subsequently affects the NVU by spatial changes. The impact of glia volume changes on neurons is facilitated and relayed by the fact that glia ensheath pre- and post-synaptic terminals of neurons to form the “tripartite synapse” (Araque et al., 1999; Santello et al., 2012; Hillen et al., 2018). Due to this physical proximity, changes in glia cell size can modulate the extracellular space and subsequently neuron excitability (Florence et al., 2012; Vecino et al., 2016). This is achieved by the synergistic activity of Aquaporin 4 (Aqp4), a channel protein, which is needed for water transport and is enriched in astrocytic endfeet (Gleiser et al., 2016), as well as the transient receptor potential cation channel TRPV4 for Ca^2+^ influx (Jo et al., 2015). However, even though Aqp4 and TRPV4 are considered main factors, glia swelling is a complex process and, depending on the context, other factors were shown to play a role in glia volume changes. These include K^+^ ion transport *via* connexin 43, Kir 4.1, or Na^+^/K^+^-ATPase, and ion flux *via* Na^+^-K^+^-Cl^–^ co-transporter (NKCC1), or glutamate movement *via* specialized transporters (Lafrenaye and Simard, 2019). These factors can also change in disease or upon injury, as exemplified by sulfonylurea receptor 1 – transient receptor potential melastatin 4 (SUR1-TRPM4) which is upregulated in CNS injury (Mehta et al., 2015), but the impact of such changes on NVU function remain poorly defined.

Besides this physical link, glia also functionally link NVU components, exemplified by their impact on EC junctions, transporters, and pathways (Figure 1; Hayashi et al., 1997; McAllister et al., 2001; Haseloff et al., 2005). This functional link is in part achieved by factors such as glial cell line-derived neurotrophic factor (GDNF), VEGF, fibroblast growth factor 2 (FGF2), or angiopoietin-1 (Figures 2B,C; Igarashi et al., 1999; Lee et al., 2003; Lécuyer et al., 2016; Blanco-Suarez et al., 2018; Jha et al., 2018). But glia cells are also key to supporting neurotransmission by removal of neurotransmitters to terminate synaptic transmission and reestablish neuronal excitability, thus avoiding toxic overstimulation, called excitotoxicity (Turner and Adamson, 2011). Glia also directly regulate neuronal activity within the synapse (Pannasch and Rouach, 2013; Sibille et al., 2014) and synchronize/modulate synaptic inputs (Fellin et al., 2004; Fellin, 2009) on the level of signaling *via* gliotransmitters, such as gamma-aminobutyric acid (GABA), glutamate, or cytokines (Kim et al., 2020). The major neurotransmitters are the excitatory glutamate and the inhibitory GABA, working together to regulate CNS function. Following the removal of neurotransmitters from the synaptic cleft, glia cells transfer these neurotransmitters back to neurons in a process called the glutamate-glutamine cycle that requires ammonia (NH_3_) and ammonium ion (NH_4_^+^) derived from NVU blood vessels (Bak et al., 2006; Limón et al., 2021), thus reestablishing functional neuron neurotransmitter pools. This maintenance of synaptic potentials comes at a very high metabolic cost with the energy for this provided by astrocytes and blood vessels (Vergara et al., 2019). While it was previously assumed that both glutamate and GABAergic neurons are under astrocytic control, a recent *in vitro* study challenges this, suggesting that GABAergic neurons establish functional synaptic transmission without glia (Turko et al., 2019). Another neuromodulator released from neurons or glia (Butt, 2011), impacting neuronal function, is adenosine derived from adenosine triphosphate (ATP) breakdown. Adenosine stimulates receptors that regulate the release of GABA, glutamate, acetylcholine, noradrenaline, 5-HT, and dopamine (Sperlágh and Vizi, 2011).

Thus, glia are specialized morphologically, biophysically, and molecularly to support and regulate the NVU. However, the multitude of glial functions within the NVU makes it challenging to discern which mechanisms are necessary and sufficient for NVU form and function. To understand the role of glia cells, further studies are needed where glia cells are disrupted (i.e., lacking, inhibited, or overactive). Such studies will allow us to disentangle NVU interactions, establish the exact role of glia cells within it and the pathophysiological consequences.

## Glia Cells and the Neurovascular Unit Vasculature: Angiogenesis and Regulation of Blood Flow

As glia directly contact and ensheath blood vessels, they directly influence EC structure and function rather than passively co-exist. Indeed, in the last decade, it has become clear that glial cells play an active role in facilitating vascular angiogenesis *via* expression of factors, such as VEGF or transforming growth factor 1β (TGF-1β) in radial glia cells (Virgintino et al., 2003; Welser et al., 2010; Biswas et al., 2017; Siqueira et al., 2018). Moreover, radial glia were shown to stabilize murine nascent cortex vessels *via* inhibition of Wnt signaling and proliferation in EC in a contact- and age-dependent manner, a process which is potentially mediated by MMP-2 (Ma et al., 2013). Glia cells, specifically CNS-specific macrophages (microglia), were also shown to play pivotal roles in the fusion of blood vessels, called anastomosis. This is achieved by Notch1-expressing macrophages that link path-seeking dll4-expressing vascular tip cells (Outtz et al., 2011) as well as by acting as physical chaperones which express TIE2 and NRP1, regulating anastomosis downstream of VEGF-mediated endothelial tip cell induction (Fantin et al., 2010). After vessel- and NVU-formation, glia are also pivotal for BBB maturity, *via* factors such as retinoic acid supplied by radial glial cells, which increases BBB stability and the expression of BBB-specific genes such as p-glycoprotein (P-gp), occludin, and Glut-1 (Mizee et al., 2013) [see details for BBB transport systems and junctions (Zhao et al., 2015)]. Similarly, astrocytic Src suppressed C kinase substrate (SSeCKS) reduces VEGF and increases EC tight junctions (Lee et al., 2003), or astrocytic angiotensin-converting enzyme-1 (ACE-1), which produces angiotensin II that facilitates BBB maturation and junction protein stabilization (Lavoie and Sigmund, 2003; Wosik et al., 2007). ECs and glia interact bidirectionally in NVU development. Firstly, radial glia support EC maturation toward decreased proliferation, reduced tip cell marker DLL4, and reduced vascular permeability, thus supporting BBB maturation. Subsequently, ECs increase GFAP-positivity in radial glia in a VEGF-A dependent manner, leading to astrocyte differentiation and NVU formation (da Silva et al., 2019). In addition to these molecular impacts, the migration patterns of blood vessels, astrocytes, and neurons are closely associated with each other like scaffolds, with astrocytes providing VEGF for EC migration and, *vice versa*, ECs in turn provide oxygen for astrocyte differentiation (Bozoyan et al., 2012; Duan et al., 2017; O’Sullivan et al., 2017). Critically, in pathology, lactate-stimulated MG express G-protein–coupled receptor 81 (GPR81), which triggers neovascularization *via* pathways such as Wnt or Norrin (Madaan et al., 2019).

Besides these roles in angiogenesis, glia are also pivotal in regulating blood flow *via* regulating NVU synaptic activity as well as by releasing factors such as calcium, NO, arachidonic acid, and prostaglandins (Gordon et al., 2007; Attwell et al., 2010; Biesecker et al., 2016; Magaki et al., 2018). It was also shown that angiotensinogen-to-angiotensin II cleavage occurs in glia, with angiotensin I receptor (AT1-R) causing vasoconstriction (Kawamura et al., 2004), while AT2-R causing vasodilation (Fletcher et al., 2010). Moreover, glia contribute to vasodilation indirectly by interaction with other NVU components such as pericytes, which then, in turn, impacts the vasodilatory state (Nortley and Attwell, 2017). Here, one important molecule is calcium, with calcium signaling not only being coordinated between glia cells (Muñoz et al., 2015), but also ECs (Thakore et al., 2021). It remains to be understood to which extent glia-EC signaling is indirectly (*via* neurons or pericytes) or directly coupled. Lastly, neuron-derived NO regulates glia-mediated vasodilation *via* prostanoids and epoxyeicosatrienoic acids (Someya et al., 2019). Together, glial cells therefore not only play a role in angiogenesis, anastomosis, EC maturation, and blood flow regulation, with glia dysfunction potentially leading to BBB breakdown, pathological vascularization, dysregulation of vasoregulation and failure to deliver sufficient oxygenation.

## Reciprocal Neuronal-to-Vascular Transport *via* Glia

As the CNS has high metabolic demands and lacks a carbohydrate storage system, the NVU is critical to serving the retina and brain metabolic needs as glucose has to be continuously supplied *via* the blood to meet the constant CNS energy demand. This is particularly crucial as neurons rely on oxidative metabolism, making them sensitive to changes in levels of oxygen and potentially ischemia (Turner and Adamson, 2011); on the other hand, astrocytes rely on glycolytic metabolism, and glucose can be stored in them in the form of glycogen, rendering them central players in NVU and CNS metabolism (Öz et al., 2007). Crucially, pyruvate carboxylase, an enzyme that is key to synthesizing the neurotransmitter glutamate from glucose *via* the anaplerotic pathway, is almost exclusively expressed in astrocytes, positioning them as essential producers of the neurotransmitters GABA and glutamate (Schousboe et al., 2019). Recently it was shown that retinal MG also conduct anaplerosis (Singh et al., 2020), suggesting that MG support retinal NVU metabolism similar to astrocytes in the brain.

While the majority of vascular-to-neuron glucose metabolism occurs directly *via* glia in the NVU, a minor proportion of the glucose flux happens directly between blood vessels and neurons (Maoz et al., 2018). At the end of glycolysis, lactate and pyruvate are produced, but instead of being merely “end-products” they are utilized further to generate energy. Indeed, lactate is taken up by neurons and is metabolized in preference to glucose when both are available (Bouzier-Sore et al., 2003; Bouzier-Sore et al., 2006). Once transported into the neuron, lactate is converted to pyruvate and used for ATP production. Thus, glia not only provide compounds for neurons but also complement them in their metabolic requirements. Besides glucose metabolism, astrocytes are also crucial for fatty acid oxidation (FAO) to generate ATP, and catabolic ketogenesis to generate ketone bodies for neuronal metabolism; with ketone bodies or lactate being used by neurons as an energy substrate to produce ATP (Bélanger and Magistretti, 2009; Souza et al., 2019). Importantly, neurons produce toxic fatty acids that are endocytosed by NVU astrocytes for cytoprotection and CNS health (Ioannou et al., 2019). Metabolically, astrocytes also play a critical role in L-serine *de novo* synthesis, which is converted to D-serine in neurons, acting as a co-agonist of N-methyl-D-aspartate (NMDA) receptors (Yamasaki et al., 2001). Together, these data demonstrate that glial cells play a key role in maintaining the homeostatic status of the metabolism of neurotransmitters, glucose, FAO, L-serine, as well as NO, essential for maintaining normal function of the NVU. Any disturbance of this fine-tuned balance, therefore, such as would occur in diseases as diverse as stroke and diabetes, will influence NVU function resulting in further failure to supply adequate essential substrates for normal neuronal function.

In addition to the above metabolic coupling, neuron-to-vascular coupling is achieved by NO, a gaseous neurotransmitter acting as a vasodilator that is needed for neurovascular coupling and regulating the vasodilatory vascular response, called functional hyperemia (Hoiland et al., 2020). In hyperemia, glia cells are essential in relaying either vasodilation or vasoconstriction depending on the available NO concentration (Metea and Newman, 2007). Further to physiological NO, glia-mediated NO and gliosis-related production of reactive oxygen (ROS) or nitrogen species (RNS), play a role in nitro-oxidative stress such as in neuroinflammation and disease such as epileptogenesis. Indeed, Sharma et al. suggest that epilepsy is preceded by a cascade of reactive gliosis, ROS/RNS, inflammatory cytokines, and neurodegeneration (Sharma et al., 2019). This neuro-vascular metabolic coupling of glucose, fatty acid, L-serine, and NO *via* glia is dependent on glia directly contacting ECs within the NVU. The required spatial connections between endfeet and ECs are partially achieved *via* connexins, which form intercellular gap junction channels and hemichannels that are expressed in ECs and astrocyte endfeet, as well as are associated with BBB maturation (Zhao et al., 2018). Also, Pannexins (Panx) that resemble connexin-based hemichannels, are thought to play a role in vasculo-neuro coupling although there is still a debate on their function in CNS development and health (Giaume et al., 2020).

## Glial Cells and the Neurovascular Unit in Disease – Pathogenesis, and Dysfunction

Beyond their roles in physiological conditions, glia and microglia contribute to neuroinflammation in response to injury, stroke, or other neurological diseases (Cekanaviciute and Buckwalter, 2016). They act as primary initiators of the inflammation cascade by increased reactivity and the secretion of factors such as chemokines (Sofroniew and Vinters, 2010; Karve et al., 2016). One ubiquitous biomarker of so-called glial activation is increased expression of GFAP (Diaz-Arrastia et al., 2013). Despite the extensive list of definitions for different glia cell states, the field agrees that inactive and reactive glia cells display changes in molecular profiles, including alterations in the cytoskeleton, metabolism, chaperones, secreted proteins, signaling proteins, and transporters. These molecular changes are accompanied by morphological transformation in cellular phenotype, such as hypertrophy (Escartin et al., 2021), which may impact the ability of glial cells to provide multiple support functions for each NVU component(s) and hence its uncoupling.

Besides the physiological functions of glial cells in inflammation, their roles in pathological settings are of wider interest. For example, increasing evidence also shows glia as a link between vascular and neurological contributions in cognitive impairment, Alzheimer’s Disease (AD), and seizures (Burda and Sofroniew, 2014; Edison et al., 2018; Price et al., 2018; Diaz Verdugo et al., 2019). Neuropathologically, AD is characterized by intracellular neurofibrillary tangles and brain parenchymal amyloid β-peptide (Aβ) deposits. The latter form neuritic plaques and cerebral amyloid angiopathy, leading to angiopathy and NVU dysregulation (Soto-Rojas et al., 2021). Astrocytes have been shown to degrade amyloid-beta in an apolipoprotein E (APOE)-dependent manner, a process that could be impaired in AD (Koistinaho et al., 2004), leading to plaque formation. Neurodegenerative disorders are complex conditions with multiple underlying causes and the role of glia has not been fully elucidated (Gleichman and Carmichael, 2020). Nevertheless, several astrocyte/glia risk factor genes, such as APOE, particularly the E4 isoform (Pihlstrøm et al., 2018) or Clusterin (CLU) and FERM Domain Containing Kindlin 2 (FERMT2) (Verheijen and Sleegers, 2018), have been identified for AD. Also, it is generally acknowledged that AD involves inflammatory responses, initiated, or mediated *via* microglia and astrocytes that lead to BBB breakdown (Akiyama et al., 2000; Nagele et al., 2004). Throughout AD disease progression, different aspects were found to affect the NVU. In pre-senile AD, increased proliferation (Ki-67), gliosis (GFAP), and vascular changes, but not neurogenesis were shown (Boekhoorn et al., 2006). In late-onset AD, vascular dysregulation and BBB breakdown are considered as the earliest biomarker (Iturria-Medina et al., 2016; Sweeney et al., 2018), with vascular dysregulation preceding changes in amyloid beta deposition, metabolism, function, structure, and memory. However, future work is needed to link genetic and mechanistic causes of AD and the spatiotemporal impacts on individual NVU components and NVU function.

In the retina, understanding the NVU and glia contribution is of particular interest as NVU dysfunction can precede neural dysfunction in patient retinas, such as in patients with Type 1 Diabetes who exhibit DR (Lasta et al., 2013). In DR and other retinal diseases, inflammation and accompanying side effects are critical contributors to disease progression and vision loss. Crucially, it has been shown that MG provide VEGF, which contributes to the upregulation of inflammatory markers, such as ICAM1 and TNFα (Le, 2017). These pro-inflammatory compounds induce pathological vascular leakage and retinal neovascularization, making anti-VEGF therapies an important therapeutic strategy in DR. In DR, it was shown that the MGs that express VEGF display a distinct morphology (Pierce et al., 1995), suggesting that they enter a reactive phenotype with altered function before expressing VEGF, which in turn leads to changes in NVU and vascular function. Besides morphological changes and increased VEGF expression, MG also show altered expression of trophic factors in DR, such as NGF, brain-derived neurotrophic factor (BDNF), neurotrophin-3 (NT-3), neurotrophin-4 (NT-4), ciliary neurotrophic factor (CNTF), or glial cell line-derived neurotrophic factor (GDNF), as well as inflammatory factors such as interleukin 1β (IL-1β), IL-6, IL-8, and TNF-α. Together, these factors contribute to disease progression and neuron survival (Boss et al., 2017). Indeed, in diabetes, it is likely that such changes result in early NVU dysfunction and subsequent failure of neurovascular coupling and hence an insufficient supply of nutrients, which then cause further damage to the NVU. Another retinal disease with clear implications of MG dysfunction is macular telangiectasia 2 which causes loss of central vision due to vascular defects (MacTel 2) (Powner et al., 2010a,b). Critically, it was shown that loss of MG matches the area of macular pigment depletion in MacTel 2 patients (Powner et al., 2010b). Moreover, the loss of these MG impacts on the NVU as a secondary hallmark feature of MacTel 2 is the formation of dysfunctional telangiectatic vessels. One potentially crucial aspect when thinking about the regional restriction of MG loss and vascular defects is that macular MG rely more on serine biosynthesis than peripheral MG (Zhang T. et al., 2019), with serine synthesis disruption resulting in mitochondria dysfunction and oxidative stress and ultimately retinal pathology (Zhang et al., 2018). This retinal pathology is also associated with swelling-induced MG volume changes (Lafrenaye and Simard, 2019) and penetration of astrocytes into deeper layers (Coffey et al., 2007). Thus, rather than having a passive role in pathogenesis, the activation state of glia, together with changes in their morphology and molecular expression, is directly associated with disease progression of diseases such as DR and MacTel 2.

Finally, it is important to note that retinal demand for oxygen is even greater than that of the brain. This is especially so during dark adaptation when metabolic activity and oxygen demand is high. The configuration of the vascular supply to the retina means that during high demand the functional reserve of oxygen is minimal, even when neurovascular coupling maximizes functional hyperemia. This makes the retina extremely vulnerable to hypoxic damage and so even small alterations to the function of any component of the NVU, including MG, is likely to result in compromised retinal function.

## Future Directions and Areas of Scientific Interest

### Combining the Strengths of Models

As the NVU is a heterologous structure formed by different cell types, it is crucial to study the NVU, and the role of glial cells in it, in various models to understand how those cells integrate to form a functional NVU. Despite our increased understanding of NVU and BBB formation, our insight into the exact processes that glial cells regulate in NVU formation and function is still far from complete. This is emphasized by the fact that *in vivo* studies are still limited in experimental scope and flexibility.

As an *in vivo* model, rodents are an invaluable pre-clinical asset to study glia and their role in the NVU. However, caution has to be paid when wanting to draw direct conclusions from pre-clinical models. For example rodent glial cells are smaller and less complex than human glial cells, with human astrocytes being 2.6-fold larger, 10-fold more GFAP-positive primary processes, and relaying signals faster (Oberheim et al., 2009), suggesting that NVU metabolism and signaling dynamics are different between rodents and human. Similarly, certain glial cells are primate-specific, such as interlaminar astrocytes, and can therefore not be studied in rodents (Colombo et al., 1995; Colombo and Reisin, 2004). To complement NVU and glia studies in rodents, zebrafish have also been invaluable, particularly as NVU function and development can be studied in real-time *in vivo* and throughout the CNS (Gestri et al., 2012; Richardson et al., 2017; Angueyra and Kindt, 2018). Zebrafish characteristics, such as *ex utero* development, genetic tractability, and embryonic transparency render them a crucial asset (MacDonald et al., 2017; Richardson et al., 2017). Crucially, the site of the BBB is conserved in zebrafish and humans in capillary ECs (O’Brown et al., 2018). However, even though neurovascular coupling was shown to be conserved, it remains to be answered to which extent the NVU is truly conserved across species (Chhabria et al., 2018). Again, glia in zebrafish and mammals show differences, exemplified by the lack of typical stellate astrocytes in zebrafish (Grupp et al., 2010) as well as the fact that progenitor cells are widely maintained in the zebrafish adult CNS while being mostly transient in mammals (Than-Trong and Bally-Cuif, 2015). However, zebrafish are being increasingly used as an experimental model to contribute to the understanding of astrocyte development and function (Mu et al., 2019; Chen et al., 2020; Muñoz-Ballester et al., 2021). Importantly, zebrafish radial glia are capable of adult neurogenesis and harbor a very high regenerative capacity, making zebrafish a suitable model to study de- and re-generation (Thummel et al., 2008; Kroehne et al., 2011; Gemberling et al., 2013; Goldman, 2014; Powell et al., 2016). *In vitro*, reductionist models have also been invaluable in providing insights to study glia and their role in the NVU. However, glial cultures, originally established by dissociation and plating of brain homogenates (Booher and Sensenbrenner, 1972; McCarthy and de Vellis, 1980), lose their structural context, rendering the information obtained of limited relevance. Similarly, cultured MGs were previously shown to de-differentiate (Hauck et al., 2003; Otteson and Phillips, 2010) casting doubt on the translatability of the data to *in vivo* settings. Advancements in establishing 3D cell cultures, however, have allowed for more physiological insights into glia biology (Haycock, 2011; Watson et al., 2017). This is especially the case when considering recent NVU studies in organoids (Nzou et al., 2020) and organ-on-a-chip (Maoz et al., 2018) models. Still, in comparison to *in vivo* models, *in vitro* studies are often considered to provide limited insight into their tissue context and developmental processes only recapitulating single-timepoint and -context information (Duke et al., 2004; Helms et al., 2016).

### Manipulating the Composition and Function of the Neurovascular Unit

Our morphological and functional understanding of the NVU, through examining the molecular composition and regional specifications of the different cell types in the CNS, has also advanced significantly. This has allowed us to begin to understand the molecular profiles of cellular components of the NVU as well as their specialization and conservation across species (Cahoy et al., 2008; Roesch et al., 2008; Zhang et al., 2014; Vanlandewijck et al., 2018; Ross et al., 2020). One aspect of particular interest is to examine glia-to-EC contacts, whether this is impacted by regional specialization, and whether this and NVU function, is influenced by glia/EC identities. Understanding such specializations will provide novel insights into the integration of CNS barriers provided by the ECs, basal lamina, and glia limitans, as their specialization might cause them to respond differently to stimulation, injury, or disease and how it affects the NVU. Regional specializations and barrier properties might also elucidate new routes for drug delivery. Thus, to understand how NVU components function as a unit, it is also crucial to establish the contribution of the individual components and how different NVU cell types combine spatially and functionally together. While the latter can be achieved by careful observation, the former usually requires system changes, ideally cell-specific, to be introduced (e.g., removal, over-expression, inhibition of proteins or cells, etc.). This is exemplified by a recent study that used tamoxifen-inducible astrocyte ablation in mice, showing that astrocytes are key to maintaining the integrity of the BBB as loss of astrocytes coincided with vascular leakage and decreased EC ZO-1 expression (Heithoff et al., 2021) and BRB (Puñal et al., 2019), thus confirming early work undertaken over three decades ago (Janzer and Raff, 1987). This BBB alteration could not be rescued by other cells. Moreover, these alterations were accompanied by non-proliferative astrogliosis which, over time, limited vascular leakage (Heithoff et al., 2021). In addition, functional imaging is allowing the unraveling of NVU functions such as neuronal activity using dynamic calcium imaging (Chhabria et al., 2018) and cellular relationships following metabolic compound exchanges (Kanow et al., 2017). Similarly, optokinetic response measurements are increasingly used to measure visual acuity (Dietrich et al., 2019; Sugita et al., 2020). Linked to performing functional imaging, understanding metabolic fluxes, storage, and turnover is crucial in beginning to understand how NVU components communicate and support each other’s function and how they may be disturbed in disease. This understanding of metabolic pathways is particularly challenging as metabolites are difficult to visualize, particularly over long periods of time. Multi-modal studies using live dyes, *in vitro* designs, as well as computational modeling, will help in the understanding metabolic pathways, fluxes *via* glia, as well as direct neuron-to-vascular transport.

### *In vivo* Imaging and Objective Quantification to Understand Glial Cell Interactions

To understand the role of glia and the NVU, the correct tools are needed to visualize components with sufficient resolution to resolve subcellular structures. For this, zebrafish are particularly well suited as transgenic reporter lines exist to label as well as manipulate (i.e., upregulation, downregulation, and loss of function) each component of the NVU *in vivo*. As such, multi-transgenic reporter lines can be generated to visualize complex cell structures and interactions in the developing retina (Lieschke and Currie, 2007). Further, the function of the NVU can be visualized in real-time *in vivo* using several physiological readouts such as blood flow (e.g., red blood cell movement) or neuronal activity (calcium reporters). Elaborate integrations of NVU studies are needed to answer questions on the spatiotemporal integration of angiogenesis, barrierogenesis, gliogenesis, and NVU function. This is exemplified by recent studies (Rosa et al., 2015; Zhang R. et al., 2019) showing that neuronal activity during development impacts glial maturation at the synapse. However, it is unclear how these events may in turn influence glial support for neurons and ultimately NVU formation. Combining state-of-the-art visualization tools, examinations of NVU dynamics and functional studies *in vivo* will compliment high-resolution structural studies [e.g., electron microscopy (Willis, 2011)] and provide further insights into the roles of glia in the form and function of the NVU.

**FIGURE 3:**
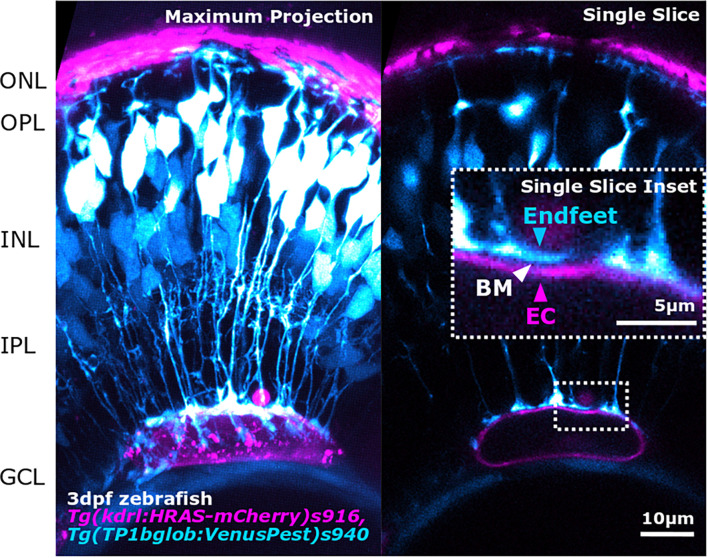
Advancements in image acquisition methods and resolution, enable the study of NVU component interactions as shown here by the interaction between MG endfeet (blue) and blood vessels (magenta), separated by the BM (white arrowhead; inset) in the developing zebrafish retina. The image was acquired with Zeiss LSM 900 AiryScan2 microscopy that allows *in vivo* acquisition with a resolution of 120 × 120 × 350 nm (x,y,z).

As glial morphology is tightly linked to their function, quantitative objective analysis of glia morphology can provide novel insights. Recent advances in NVU *in vivo* imaging, such as Two-photon microscopy (TPM), Intrinsic optical signal imaging (IOSI), Optical coherence tomography (OCT), or Laser speckle contrast imaging (LSCI) will greatly contribute to our understanding of NVU functionality and dynamics (Yoon and Jeong, 2019). This will enable imaging of functional readouts, such as blood flow, following neuronal stimuli including exposure to light flicker in the retina, to ascertain the impact of glial dysfunction on neurovascular coupling mediated events. This is complemented by ever more sophisticated tools in which to study the NVU in freely behaving animals (Cong et al., 2017; Senarathna et al., 2019) as well as the ability to investigate NVU function during development (Chhabria et al., 2018). The advancement of imaging modalities and spatio-temporal resolution offers great benefits for understanding these developmental and functional relationships in the NVU (Figure 3). However, one drawback to having this increase in resolution is the exponential increase in the amount of data that is generated per experiment. With the vast amount of experimental data, data analysis and computational expertise has become a limiting factor for many laboratories. Trained specialists and specialized data analysis training are needed to extract meaningful data in a high-throughput, standardized, and objective manner (Levet et al., 2021). Dedicated specialized computational approaches can address this analytical bottleneck to analyze data, and push scientific boundaries by computationally modeling what is experimentally impossible. This could be by *in silico* multi-transgenics (i.e., artificially overlaying transgene expression on a reference tissue) to establish a virtual retina atlas, similar to the zebrafish brain atlas, allowing for further neuronal activity mapping (Randlett et al., 2015) and co-localization analysis (Ronneberger et al., 2012) *in silico*. Similarly, deep learning approaches open new avenues from feature mining (Dollar et al., 2007), over retinal ganglion counting (Masin et al., 2021), to fundus and OCT analysis (Badar et al., 2020). Furthermore, this is accompanied by an increased assessment of feature and data connectivity and relationships, such as principal component analysis and Uniform Manifold Approximation and Projection (Allaoui et al., 2020). Additionally, carefully designed data analysis workflows will provide new insights into our data and the NVU, most likely in a way that is beyond current comprehension.

The coupling of quantitative analysis of glia shape and NVU function will also be essential in understanding the kinetics of NVU degeneration and dysfunction in disease (Luengo-Oroz et al., 2011). This is exemplified by studies that link cell feature information to genomic profiles (Yuan et al., 2012) or even link feature analysis to image cytometry, which measures cellular protein and DNA in images (Tárnok, 2006). Once robust and repeatable ways to characterize glia morphology are established, it will be possible to directly link cell morphology to function and to their molecular profiles, which could then in turn be linked to NVU functionality studies. One prominent example used transcriptomics to identify and classify retinal bipolar cells by matching their molecular expression with cell morphology (Shekhar et al., 2016). These comprehensive experimental paradigms could be used for each component in the NVU, throughout its development, to identify the molecular mechanisms regulating cell shape, function, and connectivity. These insights into precise cellular mechanisms that control the development and function of the NVU may also inform the pathogenesis of disease in mature tissues. As such, identification of the molecular and morphological changes (e.g., glial hypertrophy), that potentially precede pathology in disease, would facilitate the diagnosis of NVU dysfunction and provide the opportunity for early treatment and better clinical outcomes.

## Conclusion

It is increasingly clear that glial cells are critical for NVU development, function, maintenance, and dysfunction in disease. However, uncovering the precise contribution of glial cells to the NVU has been challenging to date, particularly when trying to understand and integrate their dual contribution to neurons and the vasculature. With the advancement of imaging, computational and genetic tools, it will be possible to use multidimensional approaches (morphology, function, genetics, interactions, and dynamics) to clarify the exact role(s) of glial cells in the NVU. Examining the role of glia in the “neuro-glial-vascular unit” with such a holistic approach will enhance the understanding, diagnosis, and treatment of aging and disease in the nervous system.

## Author Contributions

EK, JG, and RM contributed to the conception and design of the study. EK wrote the first draft of the manuscript. All authors contributed to manuscript revision, read, and approved the submitted version.

## Conflict of Interest

The authors declare that the research was conducted in the absence of any commercial or financial relationships that could be construed as a potential conflict of interest.

## Publisher’s Note

All claims expressed in this article are solely those of the authors and do not necessarily represent those of their affiliated organizations, or those of the publisher, the editors and the reviewers. Any product that may be evaluated in this article, or claim that may be made by its manufacturer, is not guaranteed or endorsed by the publisher.
